# Annotations

**Published:** 1897-12-04

**Authors:** 


### ANNOTATIONS.

### The Proposed Westminster University.

It would be difficult to find any better proof of the despair with which those who for all these years have been endeavouring to establish in London a real university regard the present position of affairs than is afforded by the fact that a serious proposal is being made to establish a University of Westminster. The manner in which London has been treated in this matter by successive Governments is undoubtedly a disgrace. In other parts of the country universities have been established and are working satisfactorily, but London, with its enormous population and its immense educational machinery is left without anything in the shape of a university in the proper sense of the term. Truly, the present University of London has done a great work in encouraging education all the world over. This, however, it has done as a mere degree-giving body open to students from all parts of the Kingdom and from the Colonies. For years back London has been trying to get for itself a teaching university—a university which should take control of the education of the student, not what the University of London is at present, a mere examining board, free to all comers, wherever trained, or whether self-trained or untrained. But what are we to say about the proposal to establish a brand new University of Westminster? As an expression of the disgust felt at the delay of all reform it may be of some use. As an illustration of lengths to which people are prepared to go rather than see the educational advantages of London wrecked by the obstructives within the present University, it may be of service. Nevertheless, we cannot consider it a step in the right direction. It partakes of the vices of other proposals, which have come to nothing; it takes for granted that the numerous bodies concerned, with all their multiplicity of interests, will co-ordinate themselves so as to constitute a university; and, more still, it takes for granted that they will work in harmony thereafter. All the work of the last dozen years tends to show that these diverse interests can only be brought into line by the authority of some outside independent body in whose judgment all parties may be expected to have confidence, and to whose decisions all parties can without loss of dignity submit, as provided for in the Bill which has twice passed the House of Lords, and only requires a little stiffbackedness in the Government to make it pass the House of Commons with the greatest ease. This Bill is in every way for the good of London; and London, with its great population ought to count for something.

### Town versus Country.

Dwellers in the country have long complained that the annual flood of town folk which every summer brings upon them carries with it many town diseases, and thus imposes a heavy penalty on the districts which are invaded. This complaint has not infrequently been made in regard to holiday homes for poor children, and now it has arisen afresh in consequence of the Maidstone epidemic, and of the attention drawn by this disaster to the possible diffusion of infectious disease by hop pickers and fruit gatherers. There is, however, another side to the question; and we can but feel that this is a case of the pot calling the kettle black, for there is but little doubt that town suffers quite as much from country as country does from town in regard to the importation of disease. It is but in the nature of things that much disease should be disseminated by means of articles of food, and so long as man lives on country products he will probably receive his share of country maladies. Neither in regard to scarlet fever or diphtheria is country milk always above suspicion; in regard to typhoid fever its disease-carrying powers are notorious; and as to tuberculosis, we can hardly avoid the belief that a good deal of disease in early life arises from imperfect regulation of milk production in the country. But of late years, with the great development of what, perhaps, we may call the craze for country holidays, country has taken to paying off town in a new fashion. People rush from what they are wont to term the fetid atmosphere of towns to partake of the sweet purity of country life. But they not infrequently get more than they ask for; and in that curious complex, that resultant of many causes, which medical officers of health plot out as the "curve" of the various diseases which come under their ken, a little hump is beginning to be noticed, indicating an increased prevalence each year soon after the time when holiday folk come back to town. "Health" resorts do not always take care that their surroundings are conducive to health; and, from what we hear, we think it not unlikely that some of the annual autumnal prevalence of typhoid fever in London is due to the consumption of typhoid-infected oysters, whelks, mussels, and other shellfish, gathered fresh from the shores over which the authorities of some of our health resorts so industriously pour out their sewage. The moral is that both pot and kettle must be cleaned, the sanitation of both town and country must be improved, for both are mutually dependent on each other.

### Education and Crime.

The *Schoolmaster* publishes some statistics which are worthy the attention of candidates for the honour of membership of a school board. In 1860 the population of England was a little over 20,000,000, and of that number 23,000, or 115 in every 100,000, were in prison. In 1870 the population had risen to nearly 23,000,000, but the proportion of it that inhabited our gaols had risen more rapidly. It was 29,000, or 128 in every 100,000. In 1872 the Education Act was passed, and in 1880, with a population of 25,000,000, the prisons contained not quite 29,000, and the proportion was 111 in the 100,000. But in these eight years the Education Act had only begun its work. Those whom it had benefited were for the most part only leaving the schoolroom; but by 1890 the whole of the younger generation were educated, and in that year the prison population had fallen to 19,624, or only 68 per 100,000 of the general population. We do not venture to say that this decrease is wholly due to the spread of education, but education opens up chances of advancement to a young man which the illiterate can never hope for, and thus it relieves the pressure in that class which is always overcrowded, because it lacks the mobility, the initiative, that education gives to the mind; not to speak of the mere knowledge of lands beyond the sea where there is room for the men who are crowded out of this little island. Education does not of itself give morality; but in spite of our occasional examples of accomplished forgers and thieves, we may claim for it that it makes morality possible for many who would otherwise have had no honest sphere open to them for the exercise of their faculties; and thus it makes indirectly for honourable living. This is what Sir George Kekewich meant when he said, "Every time I hear of another school being opened I say to myself, 'There goes another prison.'"

